# Effects of *HTR1B* 3′ region polymorphisms and functional regions on gene expression regulation

**DOI:** 10.1186/s12863-020-00886-8

**Published:** 2020-07-20

**Authors:** Xi Xia, Mei Ding, Jin-feng Xuan, Jia-xin Xing, Hao Pang, Jun Yao, Xue Wu, Bao-jie Wang

**Affiliations:** School of Forensic Medicine, China Medical University, No. 77 Puhe Road, Shenbei New District, Shenyang, 110122 China

**Keywords:** *HTR1B*, Polymorphism, Gene expression

## Abstract

**Background:**

The *HTR1B* gene encodes the 5-hydroxytryptamine (5-HT1B) receptor, which is involved in a variety of brain activities and mental disorders. The regulatory effects of non-coding regions on genomic DNA are one of many reasons for the cause of genetic-related diseases. Post-transcriptional regulation that depends on the function of 3′ regulatory regions plays a particularly important role. This study investigated the effects, on reporter gene expression, of several haplotypes of the *HTR1B* gene (rs6297, rs3827804, rs140792648, rs9361234, rs76194807, rs58138557, and rs13212041) and truncated fragments in order to analyze the function of the 3′ region of *HTR1B*.

**Results:**

We found that the haplotype, A-G-Del-C-T-Ins-A, enhanced the expression level compared to the main haplotype; A-G-Del-C-G-Ins-A; G-G-Del-C-G-Ins-G decreased the expression level. Two alleles, rs76194807T and rs6297G, exhibited different relative luciferase intensities compared to their counterparts at each locus. We also found that + 2440 ~ + 2769 bp and + 1953 ~ + 2311 bp regions both had negative effects on gene expression.

**Conclusions:**

The 3′ region of *HTR1B* has a regulatory effect on gene expression, which is likely closely associated with the interpretation of *HTR1B*-related disorders. In addition, the *HTR1B* 3′ region includes several effector binding sites that induce an inhibitory effect on gene expression.

## Background

Eukaryotic cell gene expression is usually controlled by a precise gene regulatory network. Post-transcriptional regulation plays a particularly important role in gene expression [1], including in the function of the 3′ regulatory region on mRNA. The 3′ region varies the regulatory effects with its sequence and structural characteristics, and is involved in mRNA stability, translation, and positioning. The 3′ region interacts with many kinds of effectors such as microRNAs (miRNAs) and RNA-binding proteins (RPBs) [2]. MicroRNA is a 22-nucleotide small non-coding RNA that targets a specific sequence on mRNA, mostly the 3′ regulatory region, inducing translational inhibition or mRNA degradation. More than 2000 miRNAs exist. Sixty percent of human transcriptomes are modulated by miRNAs [3]. In addition, the human genome encodes more than 1500 RBPs. The RNA-binding domains of RBPs are often involved in the recognition of the 3′ untranslated region (UTR) [4]. Variations in these regulatory mechanisms may have an impact on disease. Some diseases are more likely to be affected by genetic factors, such as observed in several mental disorders. For example, twin studies have shown that heritability of schizophrenia and autism is about 80 and 76%, respectively [5]. Compared to coding regions that only account for 1% of the human genome, the genetic associations of many diseases are more likely to exist in non-coding sequences such as the 3′ UTR [6].

Considering its extensive effects on gene expression microRNA is the primary subject of this study. It is known that miRNAs can suppress mRNA transcription or inhibit protein translation through miRNA–mRNA binding [7]. The miRNA regulatory effects are closely related to variations such as polymorphisms or mutations, which are located at miRNA target gene binding sequences. Single nucleotide polymorphisms (SNP) in miRNA target sites are known as poly-miRTS, which may produce many functional consequences. They may generate a new miRNA target site or destroy an existing one, or change miRNA–mRNA binding efficiency [8]. Many studies show that existing 3′ UTR mutations may affect the function of miRNA and lead to disease risks. In a previous study, the C allele of rs10759 significantly inhibited the binding of miR-124 to its target gene, the 3’UTR of *RGS4,* and thus affected susceptibility to schizophrenia [9]. The *H3F3B* gene strengthens its interaction with miR-616 through the s1060120 A allele, resulting in post-transcriptional suppression, and is also related to a schizophrenia phenotype [10]. A review by Sethupathy et al. also emphasized the opinion that poly-miRTS is disease related [8].

The 5-hydroxytryptamine receptor 1B gene (*HTR1B*) is associated with multiple psychiatric disorders, including schizophrenia, aggressive behavior, attention deficit hyperactivity disorder (ADHD), and substance abuse [11–13]. The length of the HTR1B gene is 3175 bp, located on chromosome 677,460,848–77,464,022 (GRCh38.p7), and contains only one exon. Based on the previous studies, several SNPs are distributed in its coding regions, such as missense mutations rs130060Cys124Phe, rs130061Phe219Leu, rs130063Ile367Val, and rs130064Glu374Lys, synonymous mutations rs6296Val287 and rs6298Ser43 [14]. In our previous study, six SNPs (rs4140535, rs1778258, rs17273700, rs1228814, rs11568817, and rs130058) were found in the 5′ region of the HTR1B gene, and other seven SNPs (rs6297, rs3827804, rs140792648, rs9361234, rs76194807, rs58138557, and rs13212041) were found in the 3′ region of the HTR1B gene [15]. This study will focus on the SNPs in the 3′ region of the HTR1B gene, which are not presented to affect any gene expression rate in eQTL GTEX Consortium data. Although a previous study has shown that the *HTR1B* rs13212041 G allele attenuates miRNA-mediated mRNA silencing [12], it is very limited in explaining the regulatory mechanisms behind these associations. In addition, extracellular miRNAs can also exist in the blood circulatory system. Most circulating miRNAs are stably present in lipid or lipoprotein complexes, such as apoptotic bodies, microvesicles, or exosomes, which act as biological markers for the diagnosis, treatment, or prognosis of complex diseases [16].

We investigated the effects of several haplotypes of the *HTR1B* gene, and truncated fragments of the 3′ region of *HTR1B,* on reporter gene expression in order to analyze the function of the 3′ region.

## Results

### The regulatory effects of polymorphisms of the 3′ region of *HTR1B*

**Fig. 1:**
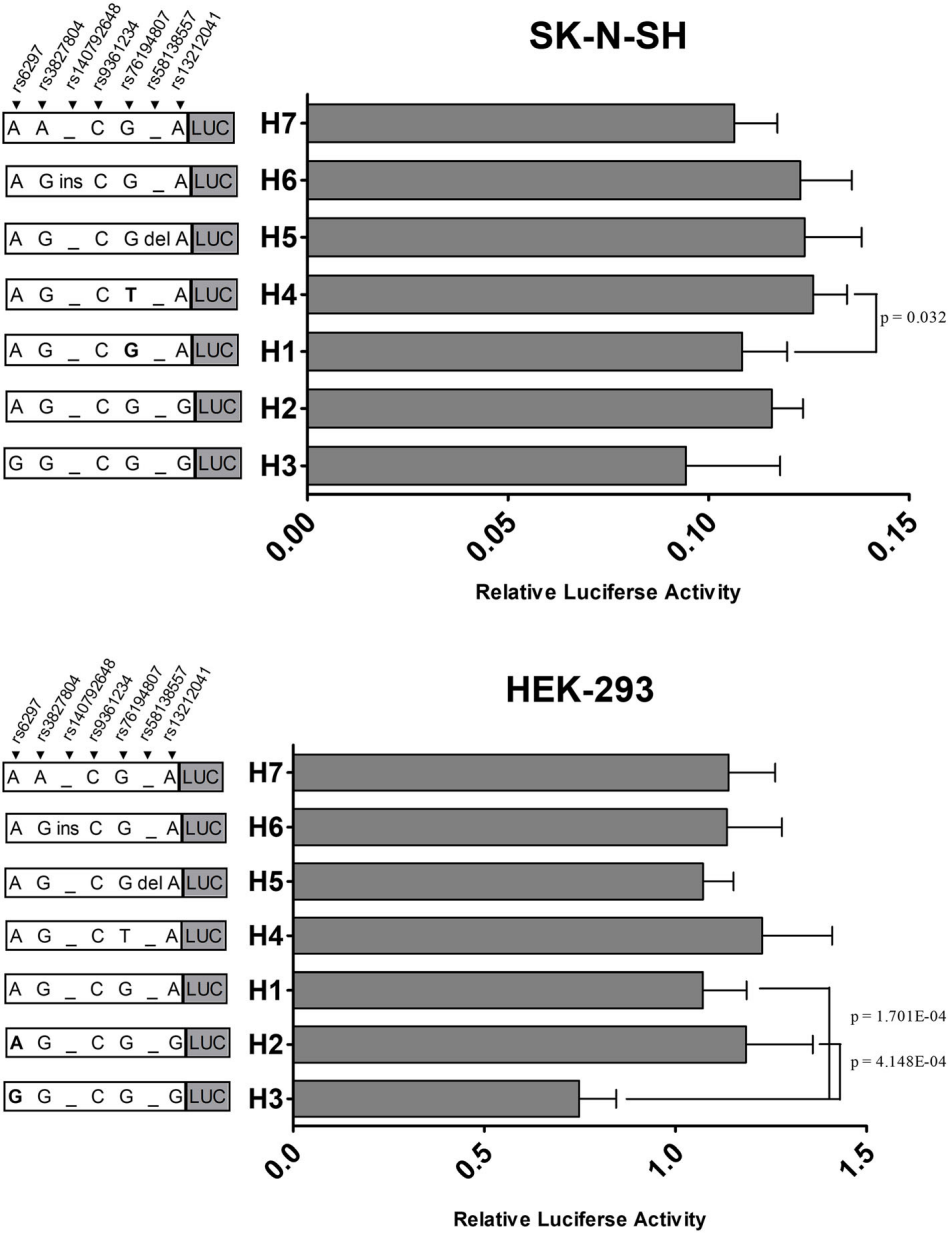
Relative fluorescence intensities of haplotype recombinants in SK-N-SH and HEK-293 cells. The haplotypes, from H2 to H7, were compared with the major haplotype, H1; the H2 haplotype was compared to the H3 haplotype. The H4 haplotype showed a higher relative fluorescence intensity than that of the HI haplotype in SK-N-SH cells. The relative fluorescence intensity of the H3 haplotype was lower than that of H1and H2 haplotypes in HEK-293 cells

Haplotype recombinants were transfected into SK-N-SH, HEK-293, and U87 cell lines. Differences in the relative fluorescence intensities among groups were present in SK-N-SH and HEK-293 cells (*P* = 3.000E-05 and *P* = 9.7158E-09, respectively), rather than U87 cells (*P* = 0.068). The data of our previous study indicated that the H1 haplotype was the major haplotype [15], having a frequency of 62.3% in the northern Han Chinese population. The H4 haplotype showed a higher relative fluorescence intensity (*P* = 0.032) in SK-N-SH cells. We noticed that for H1 and H4 haplotypes, a difference occurred at rs76194807 (G/T), suggesting that the rs76194807T allele led to an enhanced level of expression. In comparison, in HEK-293 cells we found that the relative fluorescence intensity of the H3 haplotype was lower than that of the major haplotype (*P* = 1.701E-04). In addition, a difference was also present between the relative fluorescence intensities of H2 and H3 haplotypes (*P* = 4.148E-04). Variations at rs6297 (A/G) indicated that this allele had an inhibitory effect on gene expression (Fig. 1).

### Analysis of functional sequences of the 3′ region of *HTR1B*

**Fig. 2:**
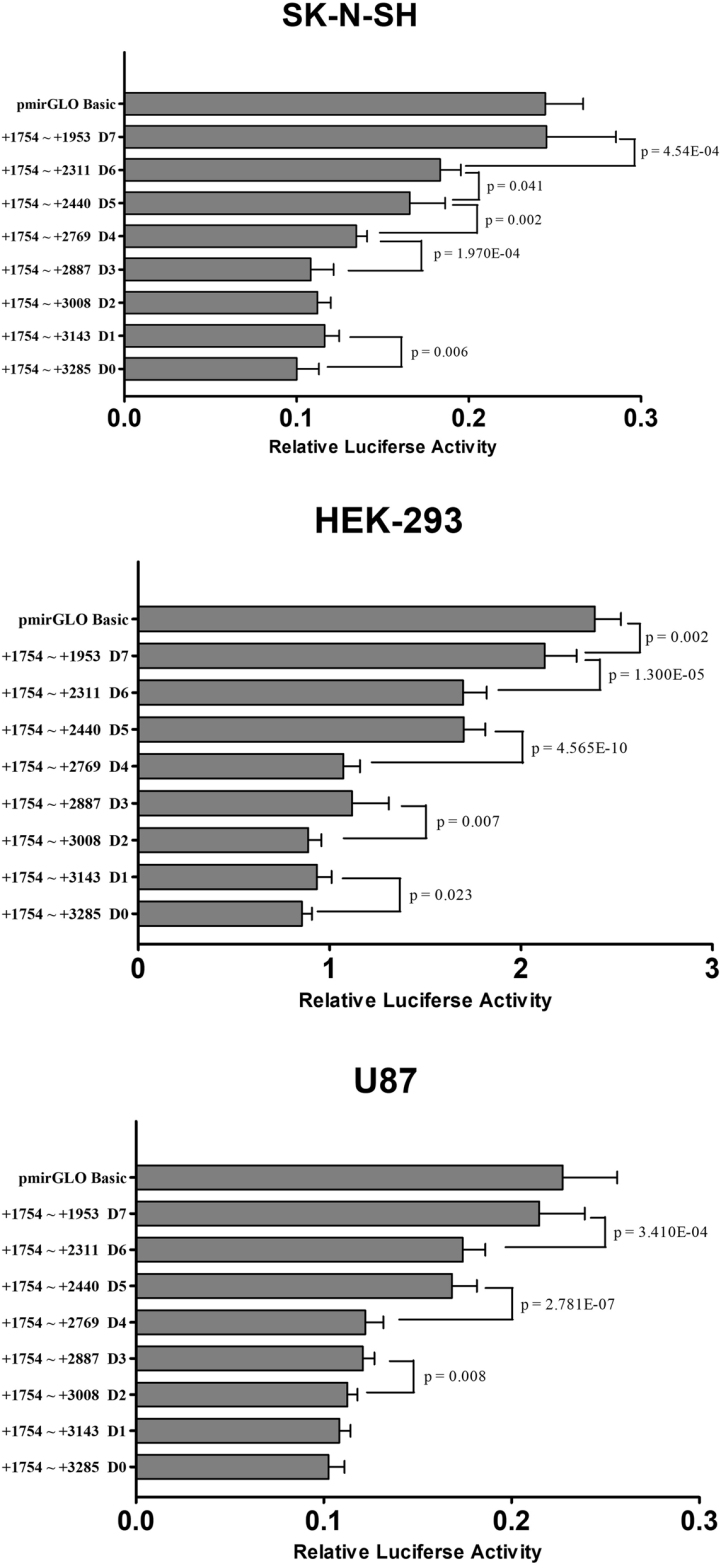
Relative fluorescence intensities of the truncated sequence recombinants in SK-N-SH, HEK-293, and U87 cells. Two regions, (+ 1754 to + 2769) versus (+ 1754 to + 2440), and (+ 1754 to + 2311) versus (+ 1754 to + 1953), showed significant regulatory effects on gene expression between in all three cell lines. Relative fluorescence intensity is expressed as the mean ± standard deviation (SD). The no-insert pmirGLO vector (pmirGLO basic) is a control plasmid

Truncated sequence recombinants were transfected into SK-N-SH, HEK-293, and U87 cell lines. Changes in the relative fluorescence intensities between the truncated fragments, D3 and D4, as well as D5 and D6, only existed in SK-N-SH cells. The truncated fragments, D0 and D1, as well as D2 and D3 showed differences in relative fluorescence intensity in two cell lines. Upward trends in the relative fluorescence intensities between the truncated fragments, D4 and D5, as well as between D6 and D7, were present in all cell lines (Fig. 2).

### Results of microRNA predictions

TarBase v.8 yields hsa-miR-21-3p, hsa-miR-941, hsa-miR-129-2-3p, hsa-miR-16-5p, hsa-miR-182-5p, and hsa-miR-26a-5p, which could target *HTR1B*. All these results were derived from high throughput experiment method.

## Discussion

The functional characteristics of the 3′ region of *HTR1B* are closely related to the interpretation of *HTR1B*-associated studies. In SK-N-SH cells, the H4 haplotype and the rs76194807T allele led to a higher level of gene expression, but previous studies have not been undertaken on either the function of rs76194807 or its association with disease. In HEK-293 cells, the H3 haplotype showed the lowest relative fluorescence intensity, and significantly suppressed gene expression compared to all other haplotypes. We also found that rs6297 (A/G) is functional, with this allele having a negative regulatory effect. This suggested that rs6297 and a potentially schizophrenia-associated polymorphism, rs1778258, had a moderate linkage relationship [15]. Researchers also found that the A-A-A-C haplotype of rs6297, rs130058, rs1213366, and rs1213371 had a potential relationship with schizophrenia in the Spanish population [11]. The rs6297G allele is also associated with susceptibility to Raynaud’s disease in an occupationally exposed Han Chinese population [17]. Therefore, the effect of rs6297 on gene expression is probably related to the mechanisms of these genetic-related diseases.

This study also investigated the functional sequence in the 3′ region of *HTR1B* using truncated fragments in order to narrow the targets that were potential binding sequences to effectors. The truncated sequence recombinants were transfected into SK-N-SH, HEK-293, and U87 cell lines. Each fragment was compared to a shorter one to identify the regulatory effect of the deleted region. It was found that truncated fragments D0 and D1, D2 and D3, D4 and D5, and D6 and D7 showed differences in at least two different cell lines. In particular, the truncated fragments D4 and D5, and D6 and D7, exhibited upward trends in all three cell lines. Therefore, the deleted sequences + 2440 ~ + 2769 bp (D4 versus D5), and + 1953 ~ + 2311 bp (D6 versus D7) are potential effector-binding regions. In addition, Jensen et al. found that miR-96 could interact with rs13212041,[9] which was located in deleted fragment + 2440 ~ + 2769 bp (D4 versus D5). However, we did not find a similar effect in this study. Since SK-N-SH, HEK-293, and U87 cell lines were used in our study while a different cell line, HeLa, was used by Jensen et al. [12], the inconsistency in results is probably derived from the tissue-specific regulation of miRNA. It also indicates that other regulatory elements other than miR-96 may exist in the + 2440 ~ + 2769 bp region to inhibit the expression of *HTR1B*. We also observed that in SK-N-SH cells, rs76194807 T and G alleles had different effects on expression level. rs76194807 is located at + 2129 bp, which happened to within the effective region of + 1953 ~ + 2311 bp. rs6297 A and G alleles are also involved in changing the gene expression level in HEK-293 cells. rs6297 is located at + 1802 bp, which is included in the effective region of + 1754 ~ + 1953 bp (Fig. 2). The mechanism underlying the potential effector-binding regions could be associated with the functional polymorphisms of the *HTR1B* gene.

Based on our results, all the truncated regions that caused the changes showed the down-regulating effect to gene expression, and as far as we know, the most classic mechanism of microRNA is to bind to the 3′ UTR region and mediate the suppression of gene expression. Predicting miRNAs binding to *HTR1B,* we found that starBase and RNAHybrid yielded miR-96 [12]. The function of miRNAs on gene regulation is very extensive. A miRNA can act on multiple genes, and one gene is usually modulated by several miRNAs. TarBase v8 predicted multiple miRNAs that could target *HTR1B*, including miR-21-3p, miR-941, miR-129-2-3p, miR-182-5p, and miR-26a-5p. Liu et al. found that miR-21 in peripheral blood is a highly sensitive and specific biomarker for the diagnosis of schizophrenia [18]. MicroR-21 was also significantly decreased in the peripheral blood of schizophrenic patients after antipsychotic treatment [16]. Transgenic mice overexpressing miR-26a-2 in serotonergic neurons displayed improved behavioral resiliency to social defeat while mice with miR-26a-2 knockdown in serotonergic neurons were more likely to be anxious. MicroR-26a-2 in the mouse dorsal raphe nucleus was also increased after antidepressant therapy [19]. Currently, no evidence exists that *Homo sapiens* (hsa)-miR-129 is associated with mental disorders, but it is closely related to the occurrence and progression of cancers [20]. MicroR-96 has been shown by Jensen et al. to have an association with aggressive behavior through interaction with *HTR1B* [12]. Hsa-miR-96 is also linked to hsa-miR-182 and hsa-miR-183, which are co-located on chromosome 7 and constitute a miRNA cluster. MicroR-183, miR-96, and miR-182 belong to the miR-183 family and are highly conserved. The previous study found that rs2402959 and rs6965643 at the miRNA 96–182-183 cluster correlated with ADHD and substance use disorders [21]. rs76481776 polymorphism caused variant pre-miR-182 associated with the dysregulation of circadian rhythms in patients with major depression and insomnia [22]. In another study that used addiction as a model of synaptogenesis and learning, injections of nicotine, cocaine or amphetamine caused changes in miRNA expression throughout the brain; miR-182 showed increased expression in the midbrain with all three drugs. This suggested that miR-182 may be involved in the regulation of addictive pathways and synaptogenesis [23].

## Conclusions

*HTR1B* 5′ regulatory region polymorphisms have regulatory effects on gene expression, and + 2440 ~ + 2769 bp and + 1953 ~ + 2311 bp regions are potential effector-binding regions that both suppress gene expression. However, further research is needed on the functional effectors binding to *HTR1B* and their association with diseases.

## Methods

### Samples

We selected genomic DNA samples, based on our previous study [15], containing seven haplotypes (consist of rs6297, rs3827804, rs140792648, rs9361234, rs76194807, rs58138557, and rs13212041), to construct pGL3 recombinants. The haplotype H1 is the major haplotype which has the highest frequency (Table 1). The target gene was obtained from GenBank using the reference sequence, NC_000006.12 (*Homo sapiens* chromosome 6, GRCh38.p7 Primary Assembly).

**Table 1:** Comparison of single nucleotide polymorphisms in haplotype recombinants

Haplotype	Frequency	rs6297	rs3827804	rs140792648	rs9361234	rs76194807	rs58138557	rs13212041
H1	0.481	A	G	Del	C	G	Ins	A
H2	0.127	A	G	Del	C	G	Ins	G
H3	0.126	G	G	Del	C	G	Ins	G
H4	0.123	A	G	Del	C	T	Ins	A
H5	0.095	A	G	Del	T	G	Del	A
H6	0.026	A	G	Ins	C	G	Ins	A
H7	0.013	A	A	Del	C	G	Ins	A

### Construction of haplotype recombinants

DNA samples containing haplotypes H1 to H7 were used as DNA templates for amplification. The target fragments were from + 1754 bp to + 3285 bp (transcription start site [TSS], + 1), including the whole 3′ UTR. The 5′ end of the primers had introduced XhoI and XabI cleavage sites. The primer sequences were 5′-CCGCTCGAGAAACTGATACGTTTTAAGTG-3′ (sense), and 5′- GCTCTAGAGCGTTTCCTGATTGTTAGTAAGTC −3′ (antisense). The purified target fragments were cloned into the pBM20S vector (Biomed, Beijing, China), and then re-cloned into the XhoI/XabI site of the pmirGLO reporter vector (Promega, Madison, WI, USA). The target haplotype of each construct was verified by DNA sequencing.

### Construction of truncated sequence recombinants

**Fig. 3:**
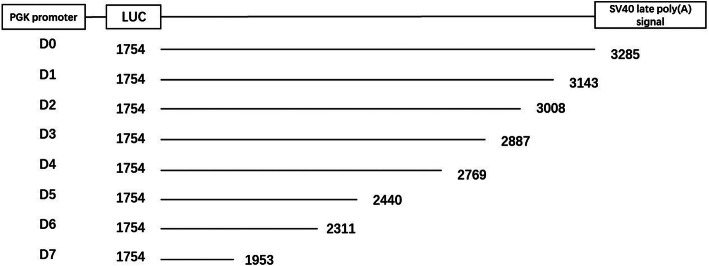
Truncated sequence recombinants in the 3′ regulatory region of *HTR1B.* The target gene was obtained using the GenBank reference sequence NC_000006.12 (*Homo sapiens* chromosome 6, GRCh38.p7 Primary Assembly). The DNA fragments shared a common 5′ end at + 1754 bp (TSS, + 1)

The target sequences were a series of DNA fragments with a common start at + 1754 bp (TSS, + 1). The longest fragment was from + 1754 to + 3285 bp, and the shortest fragment was from + 1754 to + 1953 bp (Fig. 3). The primers used for PCR are shown in Table 2. XhoI and XabI cleavage sites were introduced into the 5′ end of the primers. Truncated sequence recombinants were constructed and verified in the same manner as for the haplotype recombinants.

**Table 2:** Primer sequences of the target fragments

Primer	Length	Sequences
Common end (+ 1754)-F		5′ CCGCTCGAGAAACTGATACGTTTTAAGTG 3’
D0 (+ 1754 ~ + 3285)-R	1532 bp	5′ GCTCTAGAGCGTTTCCTGATTGTTAGTAAGT 3’
D1 (+ 1754 ~ + 3143)-R	1390 bp	5′ GCTCTAGAGCTTCTCCTGCCCAAACTTC 3’
D2 (+ 1754 ~ + 3008)-R	1255 bp	5′ GCTCTAGAGCTATGTTAGCACACAAGGAATC 3’
D3 (+ 1754 ~ + 2887)-R	1134 bp	5′ GCTCTAGAGCCCATTCCTCAATTGTGTAAG 3’
D4 (+ 1754 ~ + 2769)-R	1016 bp	5′ GCTCTAGAGCTCGGTTTTACCAATTGCAT 3’
D5 (+ 1754 ~ + 2440)-R	687 bp	5′ GCTCTAGAGCTCTCAGCATCAGAATTTTG 3’
D6 (+ 1754 ~ + 2311)-R	558 bp	5′ GCTCTAGAGCAATGATGCCAAAGTAACTGTT 3’
D7 (+ 1754 ~ + 1953)-R	200 bp	5′ GCTCTAGAGCTATTCTGGCTTCTCAGGATC 3’

### Cell lines and cell culture

Human neuroblastoma (SK-N-SH), embryonic kidney (HEK-293), and glioma (U87) cell lines [24, 25], obtained from the Cell Bank of the Chinese Academy of Sciences (Shanghai, China), were cultured in a humidified 37 °C environment at 5% CO_2_. SK-N-SH cells were grown in HyClone® DMEM supplemented with 15% fetal bovine serum (FBS; PAN-Biotech, Aidenbach, Germany). HEK-293 and U87 cells were grown in KeyGEN BioTECH® DMEM in the presence of 10% FBS.

### Transfection and dual-luciferase reporter assay

Cells were inoculated into 24-well plates (2 × 10^5^ cells/well) and grown to 90% confluence. Transient transfection was performed using Lipofectamine®3000 reagent (Invitrogen, Carlsbad, CA, USA), according to the manufacturer’s protocol. Cell lysates were collected for reporter assay 28 to 30 h post-transfection using a Dual-Luciferase® Reporter Assay System (Promega, Madison, WI, USA).

### microRNA target sites predictions

MicroRNA target sites predictions were carried out using a TarBase prediction platform [26].

### Statistical analysis

Firefly luciferase activity was divided by that of Renilla luciferase activity (LUC/TK) to yield the relative luciferase intensity. All groups of data were verified to follow a normal distribution. Differences in relative fluorescence intensity between each two haplotype recombinants were calculated using Bonferroni or Dunnett’s T3 test (based on the result of Levene’s test) following one-way analysis of variance. Differences in the relative fluorescence intensities between truncated sequence recombinants (D0 versus D1, D1 versus D2, D2 versus D3, D3 versus D4, D4 versus D5, D5 versus D6, D6 versus D7, D7 versus pmirGLO basic vector) were calculated using the independent-samples *t*-test. Calculations were carried out by SPSS22 software (IBM, Armonk, NY, USA). *P* < 0.05 indicated statistical significance.

## Data Availability

The datasets used in this study are available from the corresponding author upon reasonable request.

## Abbreviations

HTR1B: Serotonin receptor 1B
5-HT1B: 5-hydroxytryptamine receptor 1B
SNP: Single-nucleotide polymorphism
LD: Linkage disequilibrium
UTR: Untranslated region
ADHD: Attention deficit hyperactivity disorder
TSS: Transcription start site
